# Plasma Aβ analysis using magnetically-labeled immunoassays and PET ^18^F-florbetapir binding in non-demented patients with major depressive disorder

**DOI:** 10.1038/s41598-018-21140-3

**Published:** 2018-02-09

**Authors:** Kuan-Yi Wu, Ing-Tsung Hsiao, Chia-Hsiang Chen, Chia-Yih Liu, Jung-Lung Hsu, Sheng-Yao Huang, Tzu-Chen Yen, Kun-Ju Lin

**Affiliations:** 1 Department of Psychiatry, Chang Gung Memorial Hospital and Chang Gung University, Tao-Yuan, Taiwan; 2 Department of Nuclear Medicine and Center for Advanced Molecular Imaging and Translation, Chang Gung Memorial Hospital, Tao-Yuan, Taiwan; 3 Department of Medical Imaging and Radiological Sciences and Healthy Aging Research Center, Chang Gung University, Tao-Yuan, Taiwan; 4 Department of Neurology and Dementia Center, Chang Gung Memorial Hospital and Chang Gung University, Tao-Yuan, Taiwan; 5 Graduate Institute of Humanities in Medicine and Brain and Consciousness Research Center, Taipei Medical University, Taipei, Taiwan

**Keywords:** Diagnostic markers, Depression, Molecular medicine

## Abstract

An increased level of brain amyloid deposition and a decreased level of cerebral spinal fluid (CSF) Aβ42 are currently considered reliable biomarkers of Alzheimer’s disease (AD); however, the usefulness of plasma Aβ levels are not well-established. This study investigated the relationships between plasma Aβ levels and cerebral amyloidosis in 36 non-demented patients with major depressive disorder (MDD). All participants underwent ^18^F-florbetapir PET imaging and provided a blood sample at the same time for immunomagnetic reduction assay to measure the plasma levels of Aβ40 and Aβ42. We found inverse associations of the plasma Aβ42 level and the Aβ42/Aβ40 ratio, and a positive association of the plasma Aβ40 level, with cerebral amyloid deposition in the precuneus, parietal and posterior cingulate cortex. Subgroup analyses in subjects with higher ^18^F-florbetapir uptake values or MDD with amnestic mild cognitive impairment revealed more pervasive relationships of plasma Aβ measures with ^18^F-florbetapir binding across the brain regions examined. The study suggested that regional brain amyloid deposition in terms of ^18^F-florbetapir PET uptake had weak-to-moderate associations with plasma Aβ42 and Aβ40 levels, and the Aβ42/Aβ40 ratio. Validation in a larger population of subjects of known cerebral amyloidosis status is needed. Careful interpretation of plasma data is warranted.

## Introduction

Senile plaques, composed mainly of beta-amyloid (Aβ) peptides, are one of the key pathological characteristics of Alzheimer’s disease (AD). Aβ peptides indicate molecular pathogenetic events in the brain, although the presence of brain amyloid pathological changes is not sufficient to cause clinical symptoms, and is not limited to the pathology of AD. Growth of insight into pathogenic events and the course of AD has led to the establishment of new research and diagnostic criteria^1^. These criteria were recently developed by the International Working Group (IWG)^2,3^ and the task force of the National Institute on Aging and the Alzheimer Association (NIA-AA)^4^, mainly for research purposes.

The key difference between the previous and new criteria is the incorporation of reliable biomarkers into clinical symptoms, moving from the conventional clinicopathological approach to the current clinicobiological entity^1^. According to the diagnostic guidelines recommended by the IWG and the NIA-AA working group, the core amyloid biomarkers are determined by amyloid positron emission tomography (PET) and/or Aβ peptide levels in cerebrospinal fluid (CSF). Validated amyloid PET reveals *in vivo* cerebral amyloid depositions as more objective amyloid-positive evidence for the diagnosis of AD and related conditions. A decreased level of Aβ42 in the CSF is consistently considered a reliable amyloid biomarker of AD^5^.

However, amyloid imaging has a relatively high cost and limited availability in reality, especially among general practitioners^6^, and CSF Aβ samples are collected via the invasive protocol of a lumbar puncture. Therefore, there is a clear need to search other body fluids for objective biomarkers that can be used to diagnose AD^7^. Blood samples are easily obtained and are still considered potential sources of biomarkers in the clinical setting^8^.

A number of studies have focused on Aβ-related proteins in plasma. For a long time, enzyme-linked immunosorbent assay (ELISA) has been the standard and most common method of measuring the levels of Aβ-related proteins and derivatives in plasma; however, conflicting results regarding the use of ELISA to measure plasma Aβ-related proteins have been reported. Some studies found that elevated levels of plasma Aβ42 and Aβ40 might be related to the development of AD, while others reported a reduced Aβ40 level or no association in AD patients^9,10^. In addition, plasma Aβ42/Aβ40 ratio was regarded as a promising biomarker compared to individual Aβ peptide because the plasma Aβ42 level would decline as selective brain Aβ42 deposition occurs initially, and this would make the Aβ42/Aβ40 ratio fall into the lower value^11^. Nevertheless, decline of the plasma Aβ42/Aβ40 ratio has been reported in some studies, while others have reported the opposite result, or no difference^10^. A recent meta-analysis showed that the level of plasma Aβ42 did not vary significantly between AD patients and controls and cannot serve as a reliable marker for the diagnosis of AD^12^. There are several potential causes of contradictory measurements of plasma Aβ levels. First are technical difficulties: plasma Aβ42 might exist at a very low level beyond the limit of quantitation of ELISA (approximately 50 pg/ml)^13,14^. Also, quantification of Aβ might be interfered with by other plasma proteins such as albumin, lipoproteins, and complementing factors^15^. Second, study samples are heterogeneous across a wide range of different levels of cognitive impairment. The reasons described above at least partially account for controversial results in plasma Aβ measurements. Taken together, plasma Aβ proteins have not been proven to be reliable and useful biomarkers to date.

Recent advances have been made in new techniques that differ from conventional ELISA, which have been demonstrated to quantify plasma Aβ-related proteins at very low levels^16,17^. These ultra-sensitive assays seemed to provide more consistent findings about plasma Aβ data, and also provide opportunities to re-evaluate the plasma Aβ as the potential markers again. One of these assay techniques is a magnetically-labeled immunoassay, which can detect target molecules in a magnetic field by measuring magnetic signals based on immunomagnetic reduction methods (IMR)^8,18^. Some clinical studies have demonstrated the feasibility and stability of using IMR to quantify plasma Aβ levels^13,19^.

In the quest to understand the underlying mechanism of AD, there is a clear need to move research into the earlier phase of the disease, particularly the prodromal or preclinical stage of AD or related conditions. Non-demented patients with major depressive disorder (MDD) have been identified as a potential population at risk of dementia; some may be identified as a suitable sample representing the prodromal or preclinical phase of AD. In this study, we used ^18^F-florbetapir uptake as a measurement of cerebral amyloidosis and examined the correlations of PET data with plasma Aβ measures in a non-demented MDD population.

## Methods

### Subjects and protocol

Patients were recruited consecutively from the geriatric psychiatric outpatients department at Chang Gung Medical Center between August 2011 and July 2015. The inclusion criteria were age > 50 years, MDD diagnosed according to the DSM-5 criteria^20^, and a clinical dementia rating (CDR) of 0 or 0.5 without functional impairment in activities of daily living. The presence of lifetime DSM-5 major depressive episodes was reviewed in a clinical interview, and available medical information was obtained from medical records and treating physicians. The exclusion criteria were definite neurologic disorders affecting brain structure (e.g., completed stroke, traumatic head injury or epilepsy), unstable medical diseases involving the heart, lungs, liver or kidneys, and alcohol or substance abuse/dependence currently or in the past one year. None of the subjects met the NIA-AA criteria for dementia due to AD^4^, the IWG criteria for typical/atypical AD or mixed dementia^2^, or the DSM-5 criteria for any type of dementia^20^. All eligible subjects underwent ^18^F-florbetapir PET, peripheral blood sample withdrawal, and cognitive function assessments; they were also assessed for clinical characteristics of lifetime major depression. The study protocol was approved by the Institutional Review Boards of the Ministry of Health and Welfare and Chang Gung Medical Center. Informed consent was obtained from all participants and/or their legal guardians. All methods were performed in accordance with the relevant guidelines and regulations.

### Cognitive assessments

Cognitive assessments in the present study included the Mini Mental Status Examination (MMSE) and the Clinical Dementia Rating (CDR), as well as assessment of episodic verbal memory using the 12-item, six-trial selective reminding test (SRT)^21^. To include patients with varying levels of cognitive function and exclude potential cases of dementia, we used three cut-off values of the MMSE score for subjects of differing educational levels in Taiwan, which had a validated sensitivity of 100% for dementia^22,23^; i.e., less than 16 for illiterate subjects, less than 21 for grade school subjects, and less than 24 for junior high school and higher education subjects. In the SRT, the total numbers of words learned in six trials recalled, and the number recalled following a 15-minute delay, were used as the original values of the episodic memory test. These values were transformed into a composite standardized z-score, generated using regression-based norms to adjust for age and educational level according to independent normative data for Taiwan^24^. MDD patients with a composite memory z-score <−1.5 were clinically classified into the subgroup of MDD with aMCI (amnestic mild cognitive impairment) in this study.

### Image acquisition

The radiosynthesis of ^18^F-florbetapir^25^ and amyloid PET data acquisition^26^ followed the same procedures as previously described. A fixed dose of 370 MBq ^18^F-florbetapir for every subject was planned for the study. During study, each 18F-florbetapir PET scan with 378 ± 18 MBq at 50 to 60 min post-injection was obtained using a Biograph mMR PET/MR System (Siemens Medical Solutions, Malvern, PA, USA). The 3-D OSEM PET reconstruction algorithm (3 iterations, 21 subsets; Gaussian filter: 2 mm; zoom: 3) with MR-based attenuation correction, scatter and random corrections, was applied to obtain PET images of a matrix size of 344 × 344 × 127 and a voxel size of 0.83 × 0.83 × 2.03 mm^3^. T1-weighted MRI images were acquired using a sagittal magnetization-prepared rapid gradient echo (MPRAGE) sequence with the following imaging parameters: Repetition Time (TR)/Echo Time (TE) = 2600/3.12 msec, TI = 900 msec, flip angle = 13°, and a voxel size of 0.5 × 0.5 × 1.1 mm for all subjects from PET/MR and for the purpose of spatial normalization.

### Image analysis

Subsequent image analysis was performed using PMOD image analysis software (version 3.3, PMOD Technologies Ltd, Zurich, Switzerland). All PET image data were spatially normalized to the Montreal Neurological Institute (MNI) MRI template^27^ using a MR-based method. Standardized uptake value ratio (SUVR) images were generated using the whole cerebellum as the reference region. Regional SUVRs were calculated for seven volumes of interest (VOIs), including the frontal, anterior cingulate, posterior cingulate, precuneus, parietal, occipital, and temporal areas^27^, and the global cortical SUVR was obtained as the average SUVR of the 7 VOIs.

### Blood sample collection and preparation

Every subject provided a 20-ml venous blood sample (K3 EDTA, lavender-top tube). Samples were collected under non-fasting conditions between 9 AM and 2 PM for the convenience of elderly patients. The blood samples were centrifuged at 3,000 × *g* for 20 minutes within half an hour of collection, and then plasma was aliquoted into cryotubes (1 ml per tube) and stored at −80 °C. Laboratory staffs were blind to the demographic, clinical and imaging data of each subject.

### IMR measurements

The reagents used to determine plasma Aβ levels in this study consisted of dextran-coated Fe_3_O_4_ nanoparticles functionalized with antibodies. Immunomagnetic reduction (IMR) assays were the method used to probe the associations of plasma Aβ levels and magnetic nanoparticles with Aβ40 and Aβ42 reagents. This technology mainly detects the percentage reduction in an alternating current (ac) that reflects the magnetic susceptibility (Xac) of a reagent due to the interactions of functionalized magnetic nanoparticles and target proteins. The percentage reductions of immunomagnetic signals are then converted to target protein concentrations using the standard curves of the respective analytes. Details of the mechanism and technology of IMR have been reported previously^8,16^.

### Statistical analysis

Data were expressed as means ± SD or an absolute number with a proportion for descriptive statistics. Spearman correlation analysis was applied to examine the correlations between plasma Aβ measures and cerebral uptake values of ^18^F-florbetapir PET in different brain regions. Significant correlations were validated using nonparametric Spearman’s rank-order correlations. Correlation analysis was also used to evaluate the correlations between the plasma Aβ measures, ^18^F-florbetapir SUVRs, and each parameter of the demographic data, clinical characteristics and cognitive tests. Multiple linear regression analysis was used to further evaluate the associations between plasma Aβ measures and ^18^F-florbetapir binding after controlling for age and educational level. A *p* value of 0.05 was defined as the threshold of statistical significance in each test.

## Results

We recruited 36 non-demented MDD patients aged 53–71 years (mean: 62.0 ± 5.2), of whom 83.3% were women. Demographic and clinical data are provided in Table 1. Six of the 36 MDD patients (16.7%) had a composite memory z-score <–1.5, and were clinically classified into the subgroup of MDD with aMCI in this study. Neither the plasma Aβ42 level, Aβ40 level or Aβ42/ Aβ40 ratio was related to age, sex or educational level, with the exception that the plasma Aβ42 level was negatively correlated with age (r = −0.419, p = 0.011). The ^18^F-florbetapir SUVR in each of the 8 regions of interest and the global uptake were unrelated to age, sex or educational level.

**Table 1:** Demographic variables, clinical characteristics and plasma Aβ protein levels of the sample.

	MDD patients (n = 36)
Age, years	62.0 ± 5.2
Gender, female (%)	30 (83.33)
Education, years	8.1 ± 3.6
MMSE score	25.5 ± 3.6
CDR 0.5 (%)	9 (25)
HAM-D score	10.4 ± 6.3
Age of onset, years	50.7 ± 8.8
Duration, years	11.4 ± 9.6
Episodes	2.0 ± 1.2
SRT total recall	−0.77 ± 1.03
SRT delayed recall	−0.90 ± 1.12
Composite memory score	−0.84 ± 1.02
Plasma Aβ42, pg/ml	16.4 ± 2.4
Plasma Aβ40, pg/ml	49.5 ± 10.3
Plasma Aβ42/Aβ40 ratio	0.4 ± 0.1

Mean ± SD. MDD: major depressive disorder; HAM-D: 17-item Hamilton Depression Rating Scale; MMSE: Mini Mental Status Examination; SRT: Selective Reminding Test.

### Relationships of plasma Aβ measures with ^18^F-florbetapir SUVRs

**Figure 1:**
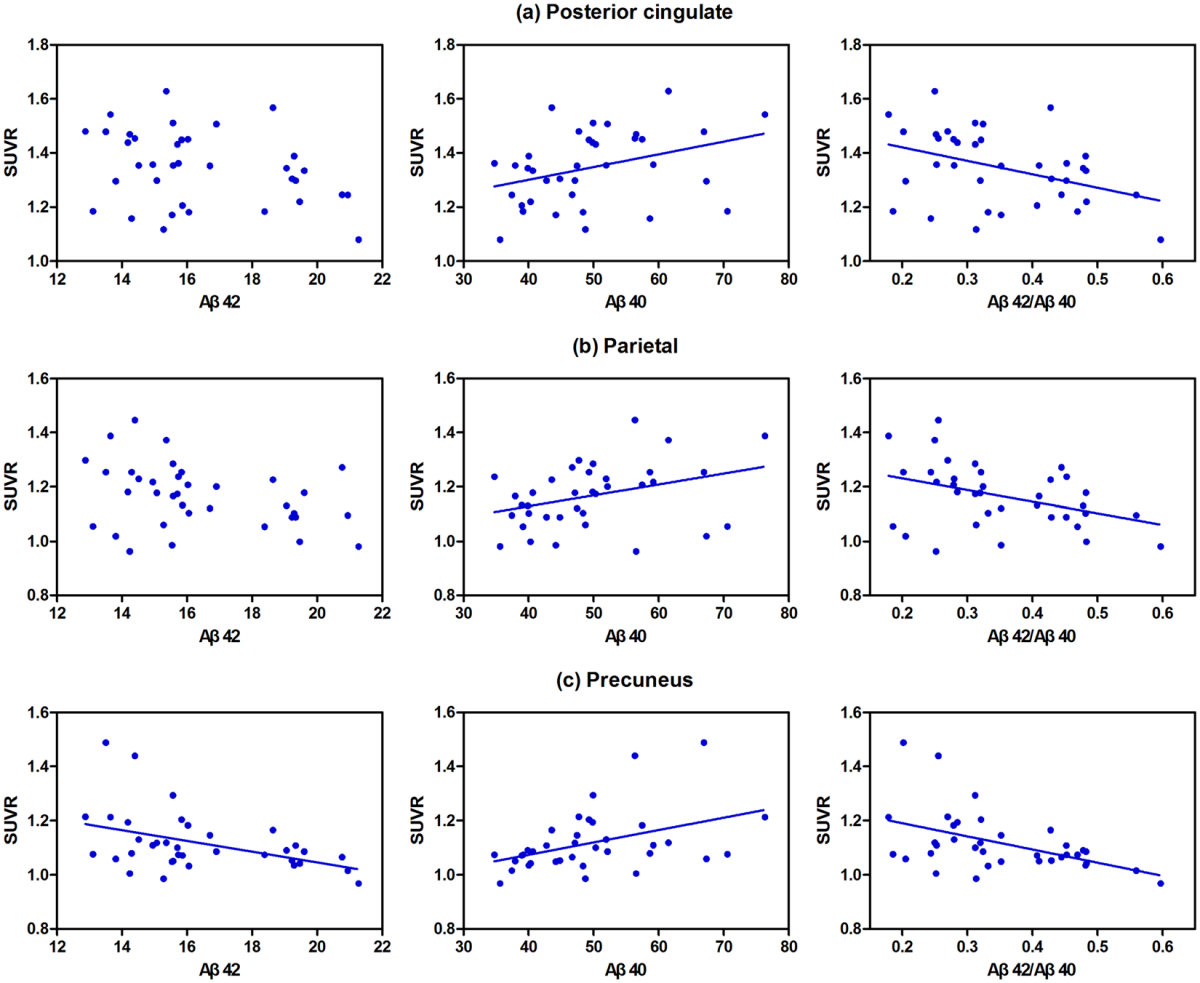
Significant correlations of ^18^F-florbetapir SUVRs and plasma Aβ42 and Aβ40 levels, and the Aβ42/Aβ40 ratio, in the (**a**) posterior cingulate, (**b**) parietal and (**c**) precuneus cortex. Regression lines represent the single lines that best fit the SUVR and plasma data.

In the whole sample, all three plasma Aβ measures were significantly correlated with the ^18^F-florbetapir SUVR in the precuneus cortex, and the Aβ40 level and Aβ42/Aβ40 ratio were significantly related to the ^18^F-florbetapir SUVRs in the parietal and posterior cingulate cortex. The plasma Aβ40 level was positively correlated with ^18^F-florbetapir uptake in the precuneus, parietal and posterior cingulate cortex (Spearman r = 0.461, 0.360, and 0.390, respectively); however, the plasma Aβ42 level was negatively correlated with uptake in the precuneus cortex (Spearman r = −0.423), and the Aβ42/Aβ40 ratio was negatively correlated with uptake in the precuneus, parietal and posterior cingulate cortex (Spearman r = −0.487, −0.401, and −0.383, respectively) (Table 2). An obvious tendency towards negative relationships of the plasma Aβ42 level and Aβ42/Aβ40 ratio with cerebral amyloid deposition, and a positive relationship between plasma Aβ40 level and amyloid burden, were consistent across all brain regions as measured by ^18^F-florbetapir uptake (Fig. 1). Further multiple regression analyses adjusting for age and educational level showed that the plasma Aβ42 level and Aβ42/Aβ40 ratio remained significantly negatively correlated with ^18^F-florbetapir uptake; the plasma Aβ40 level was positively correlated with the ^18^F-florbetapir uptake in the precuneus, parietal and posterior cingulate cortex (Table 3).

**Table 2:** Correlation coefficients between regional ^18^F-florbetapir SUVRs and plasma Aβ measures in MDD patients.

^18^F-florbetapir SUVR	MDD patients					
	Aβ42		Aβ40		Aβ42/Aβ40	
	r	p	r	p	r	p
Frontal	−0.081	0.639	0.163	0.343	−0.174	0.309
Parietal	−0.314	0.063	0.360	0.031*	−0.401	0.015*
Temporal	−0.165	0.335	0.198	0.246	−0.218	0.202
Occipital	−0.229	0.179	0.143	0.405	−0.235	0.167
Anterior cingulate	−0.083	0.631	0.104	0.547	−0.135	0.434
Posterior cingulate	−0.299	0.077	0.390	0.019*	−0.383	0.021*
Precuneus	−0.423	0.010**	0.461	0.005**	−0.487	0.003**
Global	−0.194	0.257	0.261	0.124	−0.276	0.103

MDD: major depressive disorder; *p < 0.05, **p < 0.01.

**Table 3:** Multiple regression model of ^18^F-florbetapir SUVRs and plasma Aβ42 and Aβ40 levels, or the Aβ42/Aβ40 ratio, as the predictor (adjusted for age and educational level).

n = 36	Linear regression model	
	β	p
Precuneus		
Aβ42	−0.018	0.039*
Aβ40	0.004	0.018*
Aβ42/Aβ40	−0.450	0.012*
Parietal		
Aβ42	−0.019	0.053
Aβ40	0.004	0.049*
Aβ42/Aβ40	−0.446	0.023*
Posterior cingulate		
Aβ42	−0.029	0.009**
Aβ40	0.005	0.026*
Aβ42/Aβ40	−0.636	0.004**

*p < 0.05, **p < 0.01.

### Subgroup analyses of MDD patients with ^18^F-florbetapir binding above the first quartile of the global SUVR and MDD patients with aMCI

**Figure 2:**
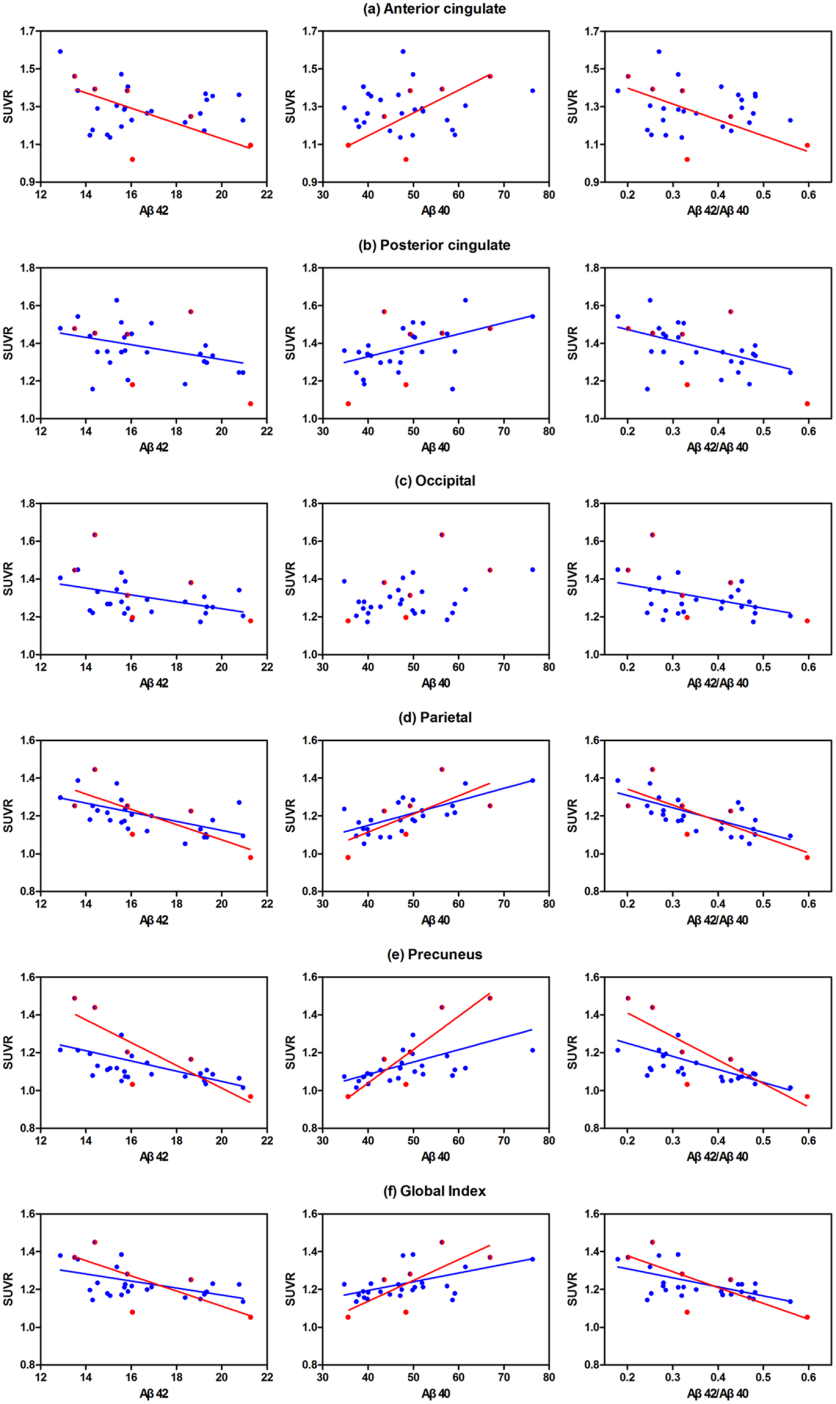
Subgroup analyses of MDD patients with a global ^18^F-florbetapir SUVR above the first quartile (blue dots) and MDD patients with aMCI (red dots). Significant correlations of ^18^F-florbetapir SUVRs and plasma Aβ42 and Aβ40 levels, and the Aβ42/Aβ40 ratio, in the (**a**) anterior cingulate, (**b**) posterior cingulate, (**c**) occipital, (**d**) parietal, (**e**) precuneus and (**f**) global cortex. Regression lines of different colors represent single lines that separately best fit the SUVR and plasma data in the two subgroups, respectively. MDD: major depressive disorder; aMCI: amnestic mild cognitive impairment.

Besides the aforementioned regions in the precuneus, parietal and posterior cingulate cortex, subgroup analysis of subjects with ^18^F-florbetapir binding above the first quartile of the global SUVR showed stronger correlations in brain regions more involved in the occipital and global cortex. All three plasma Aβ measures had more significant correlations with the ^18^F-florbetapir SUVR in the parietal and precuneus regions, and Spearman r values were more than 0.6 (p < 0.001). A second subgroup analysis performed in MDD patients who met the criteria for aMCI showed negative (Aβ42, Aβ42/Aβ40) and positive (Aβ40) correlations with the ^18^F-florbetapir SUVR in brain regions more involved with the anterior cingulate and global cortex (Fig. 2).

### Relationships between plasma Aβ measures, ^18^F-florbetapir SUVRs and cognitive function or clinical characteristics

In the whole sample, each of the three plasma Aβ measures was unrelated to MMSE score, SRT total recall, delayed recall and composite memory score. The plasma Aβ42 level and Aβ42/Aβ40 ratio were negatively correlated with disease duration (p = 0.032 and 0.048, respectively), but not with any other clinical characteristics, including HAM-D score, onset age and lifetime major depressive episodes. The ^18^F-florbetapir bindings in each of the 8 regions were also unrelated to MMSE score, SRT total recall, delayed recall and composite memory score. ^18^F-florbetapir bindings in the frontal, anterior cingulate, posterior cingulate and global cortex were negatively correlated with age of onset (p = 0.038, 0.037, 0.045, 0.049, respectively).

In the subgroup of subjects with ^18^F-florbetapir uptakes above the first quartile of the global SUVR, each of the three plasma Aβ measures was also unrelated to MMSE score, SRT total recall or delayed recall, or clinical characteristics. However, ^18^F-florbetapir binding was negatively associated with MMSE score in the parietal region and delayed recall in the precuneus region (p = 0.041 and 0.037, respectively). There was a correlational trend between composite memory score and ^18^F-florbetapir binding in the precuneus region (p = 0.049).

## Discussion

In this study, a decreased plasma Aβ42 level and a lower Aβ42/Aβ40 ratio, in addition to an increased plasma Aβ40 level, were found to be associated with increased ^18^F-florbetapir binding in specific cortex areas, such as the precuneus, parietal and posterior cingulate cortex. Further subgroup analyses showed these inverse associations of Aβ42 and Aβ42/Aβ40, and the positive association of Aβ40, with cerebral amyloid depositions in more brain regions. In the subgroups of MDD patients with a higher amyloid burden or aMCI, all three Aβ measures exhibited even more pervasive and robust correlations with ^18^F-florbetapir binding across the ROIs examined. The inverse correlations of the plasma Aβ42 level and Aβ42/Aβ40 ratio, and the positive correlation of the Aβ40 level, with ^18^F-florbetapir uptake remained significant in multiple regression analyses after adjustment for age and educational level.

A decreased CSF Aβ42 level has been adopted into the newly-published NIA-AA and IWG criteria for AD diagnosis^3,4^. Furthermore, an inverse association of the CSF Aβ42 level with amyloid deposition as measured by PiB PET has been reported^28^. However, plasma Aβ measures still elicit conflicting results, and the associations of plasma Aβ levels with cerebral amyloidosis are not well-established^29,30^. In a meta-analysis of four studies that examined plasma Aβ measures without amyloid imaging, an increase in the plasma Aβ40 level, but not in the Aβ42 level or the Aβ42/Aβ40 ratio, was found to be weakly associated with MCI conversion to AD^31^. A larger study that examined healthy elderly subjects, and did not explicitly include patients with MCI or AD, did not observe associations between plasma Aβ levels and PiB binding^32^. In that study, the authors mentioned that plasma Aβ levels below the limit of detection in several samples might have interfered with the acquired results. Another larger study, the AIBL study of aging, identified associations between PiB and plasma Aβ across their entire sample that were similar to the findings in our study, but the associations within each diagnostic group were not significant^33^. Plasma Aβ levels have been found to be highly variable in numerous studies^9,34–37^, which may, in part, imply poor validity and poor reliability of assays for plasma Aβ levels.

Recently-published articles focused on newly-developed assays such as the IMR method^16,38^ or SimoaTM digital ELISA^17^ have demonstrated ultra-sensitive technology that can be used to examine target proteins at very low levels, such as plasma Aβ proteins. Accumulating study results have gradually arrived at a consistent finding that increased cerebral amyloid deposition is accompanied by a lower peripheral Aβ42 level and a lower Aβ42/Aβ40 ratio in patients with MCI or early AD^6,39^. The present study demonstrated similar results, in that the plasma Aβ42 level showed an inverse association in the subject group of non-demented MDD patients with heterogeneous cognitive function. Furthermore, we found that the associations between ^18^F-florbetapir binding and plasma Aβ measures were more remarkable in amyloid-positive subjects (with higher ^18^F-florbetapir binding, above the first quartile of the global SUVR) and in MDD patients with aMCI. Besides, we also performed a correlation analysis for MDD patients with different age ranges: age 53–61 years (n = 17) and age 62–71 years (n = 19). The results showed patients of age 62–71 years had significant correlation of plasma Aβ measures with ^18^F-florbetapir uptakes in more brain regions than subjects of age 53–62 years (Supplementary Figure 1). Taken together, this supports the sink hypothesis that increased sequestration of toxic amyloid proteins in the brain is associated with decreases in peripheral Aβ levels.

Notably, similar to the findings of a recent study^40^, subjects with cerebral amyloidosis below the quartile uptake values of global ^18^F-florbetapir binding presented great variations in the plasma Aβ42 level and Aβ42/Aβ40 ratio. On the one hand, this finding may reflect variable plasma Aβ levels measured beyond the limitation of detection of plasma Aβ biomarkers under ultralow cerebral amyloidosis. Alternatively, on the other hand, it might imply very early alterations in Aβ kinetics that occur before detection of cerebral amyloidosis by molecular imaging. A recent review article indicated an important role of a key transporter to clear brain Aβ peptides to the peripheral circulation is low-density lipoprotein receptor–related protein (LRP1) at the blood-brain barrier. Decreased Aβ clearance might result from down-regulation of LRP1; further, the Aβ42 clearance is less efficient than the Aβ40 by LRP1^35^. These summarized results would promote brain toxic Aβ aggregations, and lead to the decreased plasma Aβ42 levels relative to plasma Aβ40.

In exploratory analyses, we found that the plasma Aβ42 level and Aβ42/Aβ40 ratio were negatively correlated with disease duration, and the ^18^F-florbetapir bindings in the frontal, anterior cingulate, posterior cingulate and global cortex were negatively correlated with age of onset. These findings seemed to provide pathological support for the observations of some previous studies, which found that that the longer the interval of onset of the first depressive episode, the greater the risk of dementia^41,42^. However, other studies have indicated that the later the onset of late-life depression, the greater the Aβ pathology^43^. It remains controversial as to whether prior depression is a true etiologic risk factor or a prodromal manifestation of dementia. In the subgroup of subjects with a global ^18^F-florbetapir SUVR above the first quartile, we found that the ^18^F-florbetapir bindings in the parietal and precuneus regions were negatively associated with MMSE score and delayed recall. Our results were in accordance with several previous findings indicating that a greater amyloid burden is correlated with lower cognitive performance in cognitively-normal older individuals^44,45^. Also, the present study was consistent with our previous findings of a negative correlation between ^18^F-florbetapir SUVR and MMSE score in a sample of MDD patients^46,47^. Importantly, region-specific brain Aβ depositions in the parietal and precuneus cortex were similar to the amyloid distribution patterns observed in MCI or early AD^48,49^. Recently, dual phase 18F-florbetapir scan demonstrate the ability of providing perfusion-like information in additional to amyloid deposition^50^. Although not performed in our study, this may be of interest to know if there is any correlation between perfusion-like information and cognitive performance.

The major strength of this study was that the plasma Aβ samples were obtained at the same time as brain amyloid measurement by ^18^F-florbetapir PET scanning. This condition meant that a simultaneous status of plasma and cerebral amyloidosis was obtained, with no time gap between plasma sampling and amyloid PET imaging, as has been the case in other studies^40^. In addition, selection of participants from non-demented MDD patients meant that at least some of them may be representative of the prodromal or preclinical phase of AD. By moving the investigation into the earlier phase and into the population at risk of AD, we might be closer to understanding the underlying mechanism of AD. Other strengths included strictly blinded analyses of plasma and PET measurements, and subgroup analyses of a subset of participants with higher ^18^F-florbetapir uptake values as a proxy of cerebral amyloid-positive group and a subset of aMCI MDD patients as a more homogeneous study population. This was the first study to report correlations of plasma Aβ levels with cerebral amyloidosis as measured by ^18^F-florbetapir PET in a population of non-demented MDD patients.

This study had several limitations. First, the sample sizes, particularly the number of subjects with aMCI, were relatively small; this would have influenced the statistical power. Second, we measured plasma Aβ levels but lacked CSF Aβ levels for the same individuals, which would have provided additional data from which to further elucidate the underlying mechanisms between central and peripheral Aβ biomarkers. Not surprisingly, a great number of Asian people, particularly older people, are rather reluctant to undergo a lumbar puncture. This real phenomenon indicates the need for bio-fluid samples other than CSF to allow a more comfortable assessment of the levels of neurodegenerative biomarkers. Third, the study participants were MDD patients in a cognitively-normal or MCI stage, but not a dementia stage. Most importantly, caution must be taken when generalizing the findings of our present report to other populations, particularly those in the stages of dementia.

Undoubtedly, obtaining biomarkers from a blood sample is more feasible and convenient. However, it does not mean that plasma Aβ biomarkers could replace the role of brain amyloid deposition measured by amyloid PET imaging for clinical or research purposes. Plasma biomarkers present a single absolute value; in contrast, imaging biomarkers contain information on both the severity and topographic extent of amyloid deposition. Notably, from the findings of our study, different subsets of patients demonstrated varying associations of plasma Aβ levels with cerebral amyloidosis. Particularly in the subset of amyloid-negative patients unveiled by amyloid PET, who exhibited plasma Aβ measures across varying levels, combining amyloid PET and plasma Aβ data may have the potential for clinical application in predicting clinical outcome, which may be more useful than employing data from PET imaging or plasma Aβ levels alone. Continued follow-up would verify future outcomes.

## Conclusion

In this study, we found that amyloid deposition in terms of ^18^F-florbetapir SUVRs in specific regions had weak to moderate associations with the plasma Aβ42 and Aβ40 levels and the Aβ42/Aβ40 ratio by magnetically-labeled immunoassay. Clinically characteristic groups of subjects, e.g., amyloid-positive or aMCI MDD patients, as the subgroup analyses suggested, demonstrated more significant associations between plasma Aβ measures and cerebral amyloidosis. A tendency towards negative relationships of the plasma Aβ42 level, Aβ42/Aβ40 ratio, and a positive relationship of plasma Aβ40 level with cerebral amyloid deposition, was apparent across all brain regions examined. External validation in a larger population of individuals of known cerebral amyloidosis status is needed.

## Electronic supplementary material

Supplementary Figure 1
