# Author Correction: Plasma Aβ analysis using magnetically-labeled immunoassays and PET ^18^F-florbetapir binding in non-demented patients with major depressive disorder

**DOI:** 10.1038/s41598-019-43800-8

**Published:** 2019-05-17

**Authors:** Kuan-Yi Wu, Ing-Tsung Hsiao, Chia-Hsiang Chen, Chia-Yih Liu, Jung-Lung Hsu, Sheng-Yao Huang, Tzu-Chen Yen, Kun-Ju Lin

**Affiliations:** 1 Department of Psychiatry, Chang Gung Memorial Hospital and Chang Gung University, Tao-Yuan, Taiwan; 2 Department of Nuclear Medicine and Center for Advanced Molecular Imaging and Translation, Chang Gung Memorial Hospital, Tao-Yuan, Taiwan; 3 Department of Medical Imaging and Radiological Sciences and Healthy Aging Research Center, Chang Gung University, Tao-Yuan, Taiwan; 4 Department of Neurology and Dementia Center, Chang Gung Memorial Hospital and Chang Gung University, Tao-Yuan, Taiwan; 5 Graduate Institute of Humanities in Medicine and Brain and Consciousness Research Center, Taipei Medical University, Taipei, Taiwan

Correction to: *Scientific Reports* 10.1038/s41598-018-21140-3, published online 09 February 2018

In the original version of this Article, Kuan-Yi Wu and Ing-Tsung Hsiao were incorrectly listed as equally contributing authors.

In addition, the Acknowledgements section in this Article was incomplete.

“We thank Avid Radiopharmaceuticals Inc. (Philadelphia, PA, USA) for providing the precursor for the preparation of 18F-florbetapir. This study was carried out with financial support from the National Science Council and the Ministry of Science and Technology, Taiwan (MOST 105-2314-B-182A-061-, MOST 106-2314-B-182-017-MY3, MOST 104-2314-B-182A-034-, NSC 101-2314-B-182-054-MY2, NSC 100-2314-B-182-041), in addition to grants from the Research Fund of Chang Gung Memorial Hospital (CMRPG3F1031, CMRPG3E2052, CMRPG3E2051, CMRPD1C0383, CMRPG3D1802, BMRP 488 and CMRPD1E0303) and the Center for Advanced Molecular Imaging and Translation.”

now reads:

“We thank Avid Radiopharmaceuticals Inc. (Philadelphia, PA, USA) for providing the precursor for the preparation of 18F-florbetapir. This study was carried out with financial support from the National Science Council and the Ministry of Science and Technology, Taiwan (MOST 105-2314-B-182A-061-, MOST 106-2314-B-182-017-MY3, MOST 104-2314-B-182A-034-, NSC 101-2314-B-182-054-MY2, NSC 100-2314-B-182-041), in addition to grants from the Research Fund of Chang Gung Memorial Hospital (CMRPG3F1031, CMRPG3E2052, CMRPG3E2051, CMRPD1C0383, CMRPG3D1802, BMRP 488 and CMRPD1E0303) and the Center for Advanced Molecular Imaging and Translation. This study was also supported by the Ministry of Health and Welfare, Taiwan (MOHW105-TDU-B-212-113020, MOHW106-TDU-B-212-113005).”

These errors have now been corrected in the PDF and HTML versions of the paper, and in the accompanying Electronic Supplementary Material.

