# Supplementary material for: Plasma Aβ analysis using magnetically-labeled immunoassays and PET 18F-florbetapir binding in non-demented patients with major depressive disorder
Source: Sci Rep. 2018 Feb 9;8:2739. doi: 10.1038/s41598-018-21140-3 (PMC5807319; doi:10.1038/s41598-018-21140-3)

Manuscript Title: Plasma Aβ analysis using magnetically-labeled immunoassays and PET ^{18}F-florbetapir binding in non-demented patients with major depressive disorder

Authors, Institution, and Affiliations:

Kuan-Yi Wu[1], Ing-Tsung Hsiao[2,3], Chia-Hsiang Chen[1], Chia-Yih Liu[1], Jung-Lung Hsu[4,5], Sheng-Yao Huang[2,3], Tzu-Chen Yen[2,3], Kun-Ju Lin[2,3] *

[1] Department of Psychiatry, Chang Gung Memorial Hospital and Chang Gung University, Tao-Yuan, Taiwan.

[2] Department of Nuclear Medicine and Center for Advanced Molecular Imaging and Translation, Chang Gung Memorial Hospital, Tao-Yuan, Taiwan.

[3] Department of Medical Imaging and Radiological Sciences and Healthy Aging Research Center, Chang Gung University, Tao-Yuan, Taiwan.

[4] Department of Neurology and Dementia Center, Chang Gung Memorial Hospital and Chang Gung University, Tao-Yuan, Taiwan.

[5] Graduate Institute of Humanities in Medicine and Brain and Consciousness Research Center, Taipei Medical University, Taipei, Taiwan

***Corresponding author:**

Kun-Ju Lin, M.D., Ph.D.

Department of Nuclear Medicine and Molecular Imaging Center,

Chang Gung Memorial Hospital,

No 5 Fuxing St, GuiShan,

333 Taoyuan, Taiwan

Tel.: +886-(3)3281200 (ext. 2625)

E-mail: kunjulin@gmail.com

Supplementary Figure 1. Correlation analyses for MDD patients with different age ranges: age 53-61 years (blue dots) and age 62-71 years (red dots). Significant correlations of ^{18}F-florbetapir SUVRs and plasma Aβ42 and Aβ40 levels, and the Aβ42/Aβ40 ratio, in the (a) posterior cingulate, (b) parietal and (c) precuneus cortex. Regression lines represent the single lines that best fit the SUVR and plasma data.

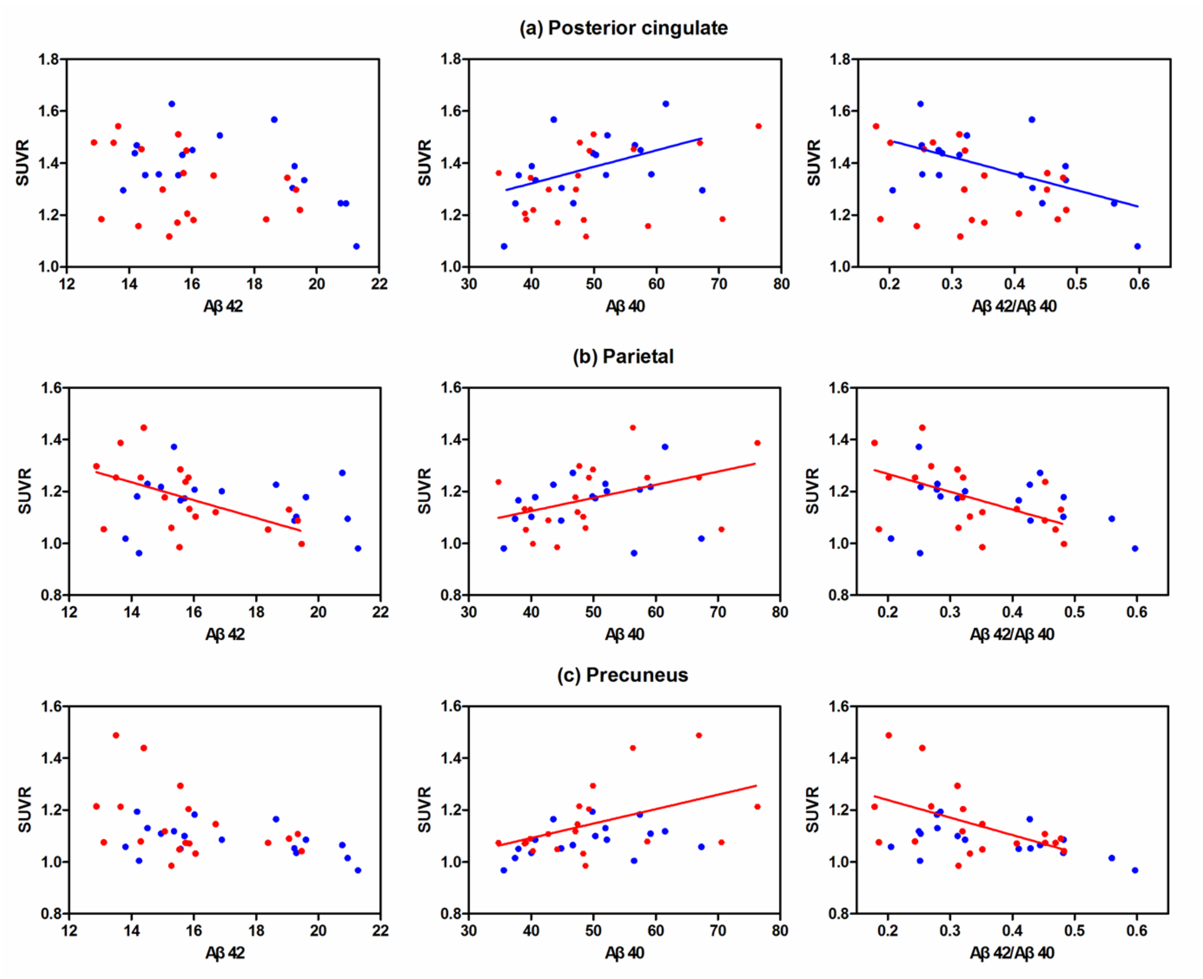

Supplement: Supplementary file 1 — Supplementary Figure 1 [file 41598_2018_21140_MOESM1_ESM.pdf]
